# Exoscopic-assisted posterior approach for the removal of thoracic dumbbell schwannoma: Three-dimensional operative video

**DOI:** 10.1016/j.heliyon.2023.e22646

**Published:** 2023-11-23

**Authors:** Francesco Restelli, Elio Mazzapicchi, Giulio Bonomo, Emanuele Rubiu, Marco Paolo Schiariti, Francesco Costa

**Affiliations:** a Department of Neurosurgery, Fondazione IRCCS Istituto Neurologico C. Besta, University of Milan, Milan, Italy; b Department of Neurological Surgery, Policlinico "G. Rodolico-S. Marco" University Hospital, Catania, Italy

**Keywords:** Dumbbell schwannomas, Ergonomics, Exoscope, Spinal tumor

## Abstract

“Dumbbell” tumors are described as benign neoplasms presenting both intraspinal and extraspinal extensions, connected through the intervertebral foramen (McCormick, 1996) [1]. About 90 % of such tumors are histologically classified as schwannomas that most frequently arise in the thoracic region (Takamura and et al., 1997) [2]. Diagnosis is usually achieved as soon as the dimensional increase of the intracanal portion results in nerve or spinal cord compression (Ishikawa and et al., 2002) [3]. How to obtain a complete surgical resection of tumors with large or ventrally located extraforaminal components with a minimally invasive approach is still debated (Payer and et al., 2006) [4]. The single-stage posterior removal of the tumor is the most performed approach for lesions presenting with a small extra-foraminal component (Payer and et al., 2006) [4]. However, due to the reduced visual surgical field and poor control of the surrounding structures that could be obtained with an operative microscopic (OM) view, the application of this approach still appears to be limited to lesions with a large extraspinal component. An alternative surgical approach is the lateral transthoracic transpleural approach, which, however, carries greater risks of complications and often requires assistance from a thoracic surgeon.

During the last decade, the exoscope was developed as a hybrid optical instrument, standing between the OM and the endoscope, merging the pros and cons of both visualization technologies, providing a wide viewing angle, high-resolution images, and non-monoaxial view.

In this work we present a case of a 60-years old male patient with a 6-month history of dorsal pain and mild left limb paresthesia resistant to conservative treatment in which for the first time a single stage exoscopic-assisted (Olympus ORBEYE 4K-3D exoscope) posterior approach was used to remove entirely a thoracic dumbbell schwannoma with large extraspinal involvement.

## Introduction Ethical compliance statement

We confirm that we have read the Journal's position on issues involved in ethical publication and affirm that this work is consistent with those guidelines.

## Consent

The patient gave written consent for participating in the procedure, surgical video and publication of her images.

## Data availability statement

Data included in article/supp. Material/referenced in the article.

## CRediT authorship contribution statement

**Francesco Restelli:** Conceptualization, Data curation, Formal analysis, Investigation, Methodology, Supervision, Validation, Visualization, Writing – original draft, Writing – review & editing. **Elio Mazzapicchi:** Conceptualization, Data curation, Investigation, Methodology, Supervision, Validation, Visualization, Writing – original draft, Writing – review & editing. **Giulio Bonomo:** Conceptualization, Data curation, Investigation, Supervision, Validation, Visualization, Writing – original draft, Writing – review & editing. **Emanuele Rubiu:** Conceptualization, Data curation, Formal analysis, Investigation, Visualization, Writing – original draft, Writing – review & editing. **Marco Paolo Schiariti:** Supervision, Validation, Visualization, Writing – original draft, Writing – review & editing. **Francesco Costa:** Conceptualization, Supervision, Validation, Visualization, Writing – original draft, Writing – review & editing.

## Declaration of competing interest

The authors declare that they have no known competing financial interests or personal relationships that could have appeared to influence the work reported in this paper.

## Abbreviations

EMG: Electromyography
MEP: Motor-Evoked Potential
MR: Magnetic Resonance
OM: Operative Microscope
OR: Operation Room
POD: Postoperative Day
SSEP: Somatosensory Evoked Potential
